# Adipose stem cells isolated from diabetic mice improve cutaneous wound healing in streptozotocin-induced diabetic mice

**DOI:** 10.1186/s13287-020-01621-x

**Published:** 2020-03-17

**Authors:** Ran An, Yong Zhang, Yu Qiao, Lili Song, Hongjun Wang, Xiao Dong

**Affiliations:** 1 College of Life Science, Qingdao Agricultural University, No. 700, Changcheng Road, Chengyang District, Qingdao, 266109 Shandong People’s Republic of China; 2 Department of Surgery, Medical University of South Carolina, Charleston, SC 29425 USA

**Keywords:** Adipose stem cells, Diabetes, Cutaneous wound healing

## Abstract

**Background:**

Adipose-derived mesenchymal stem cells (ASCs) therapy is emerging as a novel therapeutic option for the treatment of a variety of diseases including diabetes and diabetic wound healing. Multiple studies indicate that ASCs could promote wound healing and reverse diabetes. However, whether ASCs from diabetic donors retain their therapeutic functions and the mechanisms of how ASCs contribute to wound healing remain largely unknown. In this study, we explored the cutaneous wound healing ability of ASCs collected from C57BL/6 mice that had been rendered diabetic with streptozotocin (STZ).

**Methods:**

ASCs were harvested from adipose tissues of type 1 diabetic (T1D) or normal C57BL/6 mice. Cell phenotypes were evaluated by flow cytometry analysis, and cell differentiation into adipocytes, chondrocytes, and osteocytes was compared. Secretions of transforming growth factor β (TGF-β1), basic fibroblast growth factor (bFGF), and vascular endothelial growth factor (VEGF) by ASCs were assessed by ELISA. Migration and proliferation of fibroblasts co-cultured with T1D ASCs or control ASCs were also compared. The therapeutic effects of T1D and control ASCs in promoting wound closure were measured in vivo in a T1D wound mouse model. Granulation tissues were collected and stained with H&E at 14th day. CD34 and collagen I were detected by immunohistochemistry. Expressions of IL-6, α-SMA, CD31, collagen I, and collagen III were quantified by real-time PCR. GFP-expressing ASCs were used to trace in vivo cell differentiation.

**Results:**

T1D ASCs and control ASCs showed similar expression of cell surface markers (CD29, CD34, CD105) and proliferation pattern. They can both differentiate into different cell types. T1D ASCs secreted similar amounts of VEGF and bFGF, but less TGF-β compared with control ASCs. Like control ASCs, T1D ASCs promoted the proliferation and migration of skin fibroblast cells. When injected in cutaneous wound of T1D mice, T1D ASCs increased wound closure and hair follicle regeneration at a comparable extent as ASCs. Mice receiving T1D ASCs or ASCs exhibited significantly higher expressions of collagen I, collagen III, and CD31 and reduced expression of IL-6 in wound tissues. Immunohistochemistry staining showed increased angiogenesis in mice receiving ASCs as was evident by increased CD34^+^ cells and collagen I staining. GFP^+^ ASCs injection showed that ASCs differentiated into fibroblasts and endothelial cells in vivo.

**Conclusions:**

Our results suggest that T1D ASCs could accelerate cutaneous wound healing. Mechanisms may include increasing fibroblast growth and migration, skin angiogenesis, and differentiation into fibroblasts and endothelial cells. This study provides evidence that diabetic ASCs may be used as a therapeutic option in cutaneous wound healing in diabetic recipients.

## Introduction

In diabetic patients, skin can be frequently injured by acute and chronic wounds such as burning or diabetic skin ulceration, which causes physical and mental suffering in affected individuals [1]. Wound healing, an extremely complex and dynamic process, requires a cascade of various cells, growth factors, and tissue events including cellular migration, proliferation, angiogenesis, and extracellular matrix deposition [2]. Wound healing in patients with diabetes is often significantly delayed since long-term high blood glucose level causes neuropathy and impairs blood circulation. In addition, increased expression of inflammatory factors [3, 4], decreased production of growth factors and chemokines [5], proliferation and migration of fibroblasts [6], and reduced angiogenesis and secretion of collagen also contributed to delayed wound healing [7–9]. The results of current treatment options including anti-infective drugs and some physical therapy methods are far from satisfying [10, 11], so more effective treatments to expedite wound healing for diabetics are needed [12].

Bone marrow-derived mesenchymal stem cells (BM-MSCs), a subpopulation of self-renewing multipotent stem cells, have been extensively studied and proved effective in promoting skin wound healing when injected into wound site [13, 14]. However, the application of BM-MSCs is limited due to the complicated sample process and the decrease of their proliferation and differentiation capacities after in vitro culture [15] or in cells harvested from aged donors [16, 17]. Compared to BM-MSCs, ASCs can be more easily obtained in a large quantity. ASCs are similar to BM-MSCs in terms of self-renewal and differentiation capacity [18]. The growth rate, proliferation, and differentiation capacity of ASCs did not decrease after culture of several passages [19, 20]. The beneficial effects of ASCs in tissue repair have also been demonstrated [21, 22].

Compared to allogeneic cell therapy, autologous cell therapy is deemed as a safer therapeutic approach because they do not induce immune rejection responses. However, autologous cells are not always suitable for therapy since MSCs could lose their protective properties during the progression of certain diseases such as lupus [23, 24]. Diabetes is often associated with complications in multiple organ systems throughout the body, which could decrease ASC proliferation and growth factor secretion and therefore reduce their therapeutic effects [25–28]. Whether ASCs isolated from hyperglycemic donors are suitable for the treatment of cutaneous wounds remain unclear. In this study, we generated a full-thickness excisional splint wound model in diabetic mice and assessed the therapeutic effects of ASCs isolated from healthy or diabetic donors on skin wound healing. Our results show that T1D ASCs could accelerate cutaneous wound healing at a similar extent as the control ASCs.

## Material and methods

### Animals

C57BL/6J male mice at 6–8 weeks of age (Beijing Weitong Lihua Experimental Animal Technology Co. Ltd., Beijing, China) were used for this study. All animal experiments were approved by the Animal Care Committee at Qingdao Agricultural University. Tg(CAG-EGFP)B5Nagy transgenic mice used for cell tracing studies were from Dr. Wei Shen’s Laboratory (Qingdao Agricultural University).

### Induction of diabetes

Diabetes was induced in C57BL/6 mice by a single injection of STZ (i.p) (Sigma-Aldrich; St. Louis, MO, USA) at 180 mg/kg body weight. Blood glucose levels and body weights were measured daily for 7 days. Mice with two consecutive blood glucose readings above 300 mg/dl were considered as T1D mice and used for this study [29].

### Isolation and culture of ASCs

The epididymal fat tissues were collected from the C57BL/6 or STZ-induced hyperglycemic mice. Tissues were washed with phosphate-buffered saline (PBS; Sangon Biotech; Shanghai, China) and digested with 0.1% collagenase type II (Sigma-Aldrich; St. Louis, MO, USA) for 30 min at 37 **°**C. The digestion was terminated by the addition of complete culture medium that consist of DMEM/F12 (Gibco; Langley, OK, USA), supplemented with 10% fetal bovine serum, 1% pen-strep, 1% sodium pyruvate, and 1% nonessential amino acid (Gibco; Langley, OK, USA). Cells were collected and cultured in complete medium at 37 **°**C in 5% CO_2_ atmosphere. Cells were passaged when reached 90% confluence. Rates of proliferation of ASCs were measured by XTT Cell Proliferation Assay kits according to the manufacturer’s instructions (ATCC). ASCs were dispersed into 100 μl of cell suspension in 96-well plate (2000 cells/well) and cultured overnight before the addition of 10 μl cell counting solution. Cells were incubated for 1 h, and absorbance at 450 nm was measured using a microplate reader. Cells were counted daily for 7 days.

### Isolation and culture of mouse skin fibroblasts (MSFs)

Skin fibroblasts were isolated from the back skin of healthy newborn C57BL6 mice. The pieces of skin were washed with PBS and digested with 0.01% collagenase type 1 with 0.1% Trypsin (Sigma-Aldrich; St. Louis, MO, USA) for 30 min at 37 **°**C. The digestion was terminated by the addition of complete DMEM/F12 medium. Fibroblasts were collected by centrifugation and later cultured in complete DMEM/F12 medium at 37 **°**C in a 5% CO_2_ atmosphere before use.

### Characterization of ASCs

Expressions of cellular markers, including CD105, CD29, and CD34, were measured by flow cytometry analysis as described. ASCs were trypsinized and fixed in 4% paraformaldehyde, re-suspended in PBST (0.1% Tween 20 in PBS, Sangon Biotech, Shanghai, China), and then incubated with fluorescent-conjugated antibodies to CD34, CD29, and CD105 or corresponding isotype controls (Abcam; Cambridge, MA,USA) at room temperature for 30 min. Samples were analyzed with a LSR Flow Cytometer. ASCs were induced to differentiate into adipocytes, osteocytes, and chondrocytes using cell differentiation kits according to the manufacturer’s recommendations (MoBiTec, Lotzestraße, Germany). Presence of adipocytes was determined by oil red O staining, osteoblasts by alizarin red staining, and chondrocytes by alkaline blue staining as described. Expressions of Lpl, Glut-4, PPARγ, FABP-4, C/EBPα, Runx2, Alp, Opn, Sox9, Has2, and Acan at mRNA level were measured after ASCs were differentiated into adipocytes, osteocytes, and chondrocytes.

### Enzyme-linked immunosorbent assay (ELISA)

T1D or control ASCs were seeded in serum-free medium in 6-well plates (2 × 10^5^ cells per well) for 5 days. The supernatant of culture medium was then collected. Concentrations of TGF-β1, bFGF, and VEGF in cell culture medium were measured using the individual ELISA kits (Lengton Bioscience, Shanghai, China) according to the manufacturer’s instructions.

### Cell migration assay

Fibroblasts were allowed to grow till 90% confluence, and mechanical “wounds” were made on the cell culture plate by scraping with a 10-μL pipette tip. Fibroblasts were then co-cultured with T1D ASCs or normal ASCs (2 × 10^5^ cells/well) in a Transwell cell culture system (Corning; Corning, NY, USA) for 24 h. Migrations of fibroblasts were recorded at 0 and 24 h after the scratch. Percentages of field area covered by migrated cells over the total field area were determined with a computer-assisted image analysis system (ImageJ, NIH).

### Fibroblast proliferation assay

To determine the impact of ASCs from normal and diabetic mice on the proliferation of fibroblasts, fibroblasts seeded in triplicates (1 × 10^5^ cells/well) were co-cultured with control or T1D ASCs in a Transwell system. After 24 h, fibroblasts were trypsinized and the number of fibroblasts in each well was counted using a hemocytometer.

### Skin wound model and ASC treatment

Mice were anesthetized with Sumianxin II. After shaving, a biopsy punch was generated at the back of the mice to outline two circular patterns for the wound (5 mm in diameter) on each side. ASCs from T1D or control mice (5 × 10^5^, at passage 3) re-suspended in 200 μL of PBS were injected around each wound for diabetic mice. Mice injected with PBS were used as the control. Mice were housed in separated cages after treatment.

### Measurement of wound closure rate

The wounds were photographed before (0 day) and 3, 7, and 14 days post-treatment. Wound size and closure rate were calculated using the ImageJ software by the following equation: wound healing rate (%) = (*A*_o_ − *A*_t_) × 100%/*A*_o_. *A*_o_ represents the initial wound area, and *A*_t_ was the wound area at 0, 3, 7, or 14 days post-treatment.

### Hematoxylin and eosin (H&E) staining

Skin tissues were fixed in 4% paraformaldehyde for 24 h, embedded in paraffin, and sectioned. For H&E staining, the slides were immersed in filtered hematoxylin for 6 min, rinsed with water, and stained with eosin for another 1–2 min. Sections were rinsed with water and dehydrated in ascending alcohol solutions, cleared with xylene, and mounted with a cover slip for observation.

### Immunohistochemistry and immunofluorescent staining

For immunohistochemistry, tissue sections were blocked in 1% BSA for 30 min, incubated with the mouse anti-collagen I (1:200 Abcam; Cambridge, MA, USA) or rabbit anti-CD34 antibodies (1:200, Abcam; Cambridge, MA, USA) for 12 h at 4 °C. Sections were then rinsed for 3 times with buffer, followed by incubation with HRP (horseradish peroxidase)-conjugated rabbit anti-mouse or goat anti-rabbit secondary antibodies (1:200, Abcam; Cambridge, MA, USA). Slides were washed and mounted with coverslips for observation. Fluorescent-conjugated anti-CD34, GFP, and skin fibroblast actin antibodies (Abcam; Cambridge, MA, USA) were used in immunofluorescent staining. Tissue sections were blocked in 1% BSA for 30 min, followed by antibody incubation (1:1000) overnight at 4 °C. Nuclear dye DAPI was applied for 3 min at room temperature, and slides were washed and mounted with coverslips for observation. All images were captured using an Olympus IX microscope (Olympus; Tokyo, Japan).

### Quantitative real-time PCR

RNA was extracted from cells or tissues and reverse transcribed into cDNA using the Omniscript RT kit (Qiagen, Germantown, MD, USA). Gene expression was analyzed using standard protocol. PCR reactions were performed using the ABI 7700 Sequence Detection system (Applied Biosystems; Foster City, CA, USA). Fold changes in gene expression were normalized to the expression of beta actin. The following primers were used: *Lpl*: forward: CCAATGGAGGCACTTTCCAG, reverse: CCACGTCTCCGAGTCCTCTC; *Glut-4*: forward: ATGGCTGTCGCTGGTTTCTC, reverse: ACCCATAGCATCCGCAACAT; *PPARγ*: forward: ATCATCTACAATGCTGGCC, reverse: CTCCCTGGTCATGAATCCTTG; *FABP-4*: forward: GATGCCTTTGTGGGAACCTG, reverse: GAATTCCACGCCCAGTTTGA; *C/EBPα*: forward: CGCAAGAGCCGAGATAAAGC, reverse: GCCGGGTCATTGTCACTGGTCA; *Runx-2*: forward: AGTCCGCCAACTTCCTGTGCT, reverse: GGTGAAACTCTTGCCTCGTC; Alp: forward: GCCCTCTCCAAGACATATA, reverse: CCATGATCACGTCGATATCC; *Opn*: forward: AGCAAGAAACTCTTCCAAGCAA, reverse: GTGAGATTCGTCAGATTCATCCG; *Sox9*: forward: AGCTCACCAGACCCTGAGAA, reverse: TCCCAGCAATCGTTACCTTC; *Has2*: forward: TGAACAAAACGGTAGCACTCTG, reverse: ACTTTAATCCCAGGGTAGGTCAG; *Acan*: forward: ATTTCCACACGCTACACCCTG, reverse: TGGATGGGGTATCTGACTGTC; *α-SMA*: forward: TGACCCAGATTATGTTTGAGACC, reverse: CCAGAGTCCAGCACAATACCA; *collagen I*: forward: AGGCCACGCATGAGCCGAAG, reverse: GCCATGCGTCAGGAGGGCAG; *collagen III*: forward: AGGATCTGAGGGCTCGCCAGG, reverse: AGCCACCAGACTTTTCACCTCCA; transforming growth factor β (TGF-β): forward: CGCCATCTATGAGAAACC; reverse: GTAACGCCAGGAATTGT; vascular endothelial growth factor (VEGF): forward: CTACTGCCGTCCGATTGAGA, reverse: CTATGTGCTGGCTTTGGTGAG; basic fibroblast growth factor (bFGF): forward: GGCTGCTGGCTTCTAAGTGT, reverse: CCAACTGGAGTATTCCGTGAC. IL-6: forward: CCACTTCACAAGTCGGAGGCTTA, reverse: GCAAGTGCATCATCGTTGTTCATAC.

### Statistical analysis

All data shown as mean ± standard deviation (SD). Differences between groups were compared by Student’s *t* test and ANOVA test. *p* < 0.05 was considered statistical significance.

## Results

### Characteristics of diabetic mice

**Fig. 1:**
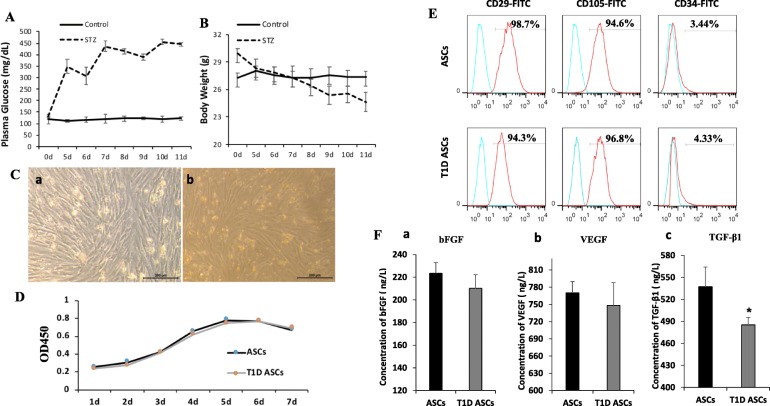
Characterization of T1D ASCs. Blood glucose levels (**A**) and body weights (**B**) of control and STZ-treated mice at different days after treatments. **C** Representative cell morphology of T1D ASCs (**a**) and ASCs (**b**) at passage 3 (bar = 200 μm). **D** Growth curves of T1D ASCs and ASCs at passage 3. **E** Cell surface antigen expression in T1D ASCs and ASCs analyzed by flow cytometry. **F** Concentration of bFGF (**a**), VEGF (**b**), and TGF-β1 (**c**) secreted by T1D ASCs and ASCs. *p < 0.05, Student’s *t* test

We first confirmed hyperglycemia of C57BL/6 mice treated with STZ. As is evident in Fig. 1A, B, blood glucose levels of treated mice were elevated at day 5 after STZ injection. The body weights of mice were significantly reduced compared to control mice receiving vehicle. Mice showed typical signs of diabetes including polyphagic, polydipsic, and polyuric. The blood glucose levels of those mice were continuously measured for 10 days to ensure they remained > 350 mg/dl before generation of the wound model. The average body weight in STZ-treated mice was 25 ± 3 g (Fig. 1B).

### Characterization of ASCs

Phenotypes of ASCs isolated from STZ-induced diabetic or control C57BL/6 mice with normoglycemia were compared. ASCs from both origins formed a monolayer with a larger spindle-shaped fibroblastic morphology during the first few days of cell culture. T1D ASCs grew slightly slower compared to control ASCs (Supplementary Fig. 1, A, B), as the control reached 90% confluence at day 7 and T1D ASCs at day 10 after initial seeding. After 3 passages, there was no significant difference in cell growth between the control and theT1D ASCs (Fig. 1C, D).

We analyzed the surface markers in T1D ASCs and ASCs by flow cytometry at passage 3. Both T1D and control ASCs were positive for CD29 (98.7% vs. 94.3%) and CD105 (94.6%; 96.8%), and negative for CD34 (3.44%; 4.33%) (Fig. 1E). T1D ASCs secreted similar amounts of bFGF and VEGF (Fig. 1F (a, b)) but significantly less amount of TGF-β1 compared to control ASCs (Fig. 1F (c)). Collectively, there were no dramatic differences on cell morphology, growth rate, cell surface marker expression, and bFGF and VEGF secretion between T1D ASCs and control ASCs.

**Fig. 2:**
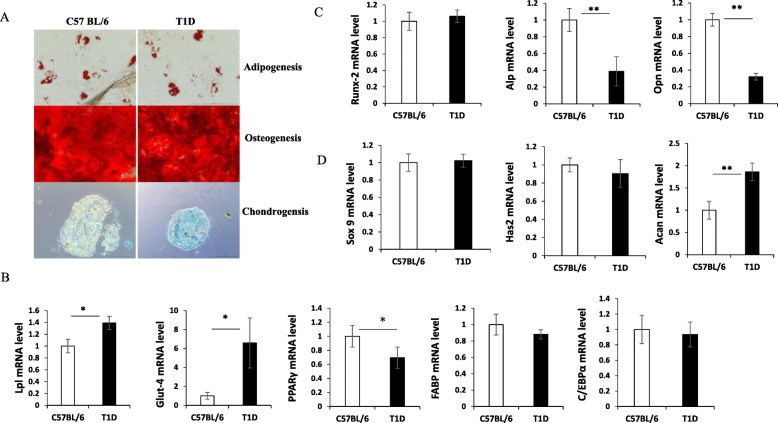
Multiple differentiation potential of ASCs harvested from control of T1D donors. **A** Representative micrographs of ASC-derived adipocytes identified by oil red staining, osteocytes by alizarin red staining, and chondrocytes by toluidine blue staining. Scale bar = 100 μm. **B** Expression of Lpl, Glut-4, PPARγ, FABP-4, and C/EBPα mRNA level in adipocytes derived from control or T1D ASCs. **C** Expression of Runx2, Alp, and Opn mRNA level in osteocytes from control or T1D ASCs. **D** Expression of Sox9, Has2, and Acan mRNA level when control and T1D ASCs were differentiated into chondrocytes. Data are from at least three individual experiments.*p < 0.05, **p < 0.01, Student’s *t* test

We compared the impact of diabetes status on lineage differentiation of ASCs isolated from control or T1D mice. We found that cells from both control of T1D mice could differentiate into adipocytes, chondrocytes, and osteocytes under standard cell induction conditions (Fig. 2A). Expressions of cell type specific genes were also measured in adipocytes, osteocytes, and chondrocytes derived from control of T1D ASCs. Our results showed that expressions of Lpl and Glut-4 were higher, while PPARγ were lower in T1D ASCs compared to control ASCs in adipocytes (*p* < 0.05). There were no differences in expression of FABP-4 and C/EBPα mRNA in those cells (Fig. 2B). Expression of Alp and Opn were significantly lower in T1D ASC-derived osteocytes compared to control ASC-derived cells (*p* < 0.01), with no change in Runx2 mRNA level (Fig. 2C). Expressions of Acan were significantly higher in T1D ASC-derived chondrocytes compared to controls (*p* < 0.01), while no difference in expression of Sox9 and Has2 mRNA level observed (Fig. 2D).

### T1D ASCs promotes wound healing in diabetic mice

**Fig. 3:**
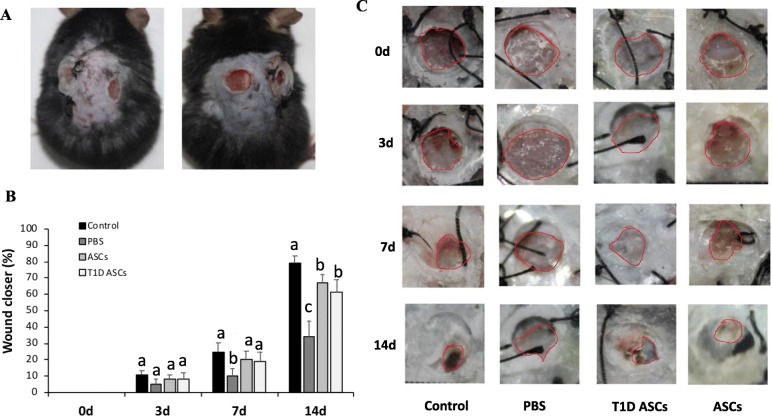
T1D ASCs promote wound healing in T1D mouse wound**. A** Representative images of excisional wound model using a donut-shaped splint to keep the wounds open. **B** Photomicrographs of wounds treated with PBS, ASCs, or T1D ASCs at 0, 3, 7, and 14 days. Control normal mice wounds were treated with PBS; T1D mice wounds were treated with PBS, T1D ASCs, or ASCs respectively. **C** The wound closure rates of T1D mice treated with PBS, ASCs, or T1D ASCs. PBS, control normal mice were treated with PBS. Date expressed as the mean ± SD; *n* = 8–10 per group; *p < 0.05, **p < 0.01 vs. PBS group, ANOVA test

Wound healing rate and wound area are reliable predictors of complete wound closure [30]. We measured wound healing rates in T1D mice receiving local injection of T1D ASCs or control (normal) ASC group in the full-thickness excisional splint wound model in diabetic mice. Non-treated C57BL/6 and T1D mice treated with PBS were used as controls. For normoglycemic control mice, the wound healing rate was 30 ± 4.5% at day 7 and 79 ± 4.0% at day 14, respectively. In contrast, T1D mice showed delayed wound healing rate, with 10 ± 3.0% wound closure at day 7 and 34 ± 5.0% at day 14. Injection with ASCs or T1D ASCs improved wound healing: at day 7 post-treatment, the wound closure rates were 20 ± 3.2% and 18 ± 3.4% in mice receiving ASCs or T1D ASCs, respectively; at 14 days post-treatment, the wound healing were 70 ± 3.8 and 60 ± 4.2%, respectively (Fig. 3B, C). Our data showed that T1D mice treated with T1D ASCs or ASCs had higher wound closure rates compared to PBS groups observed at both 7 and 14 days post-wounding. Collectively, these results indicate T1D ASCs had slightly lower but comparable protection as normal ASCs.

### T1D ASCs promote angiogenesis and collagen expression in diabetic mice

**Fig. 4:**
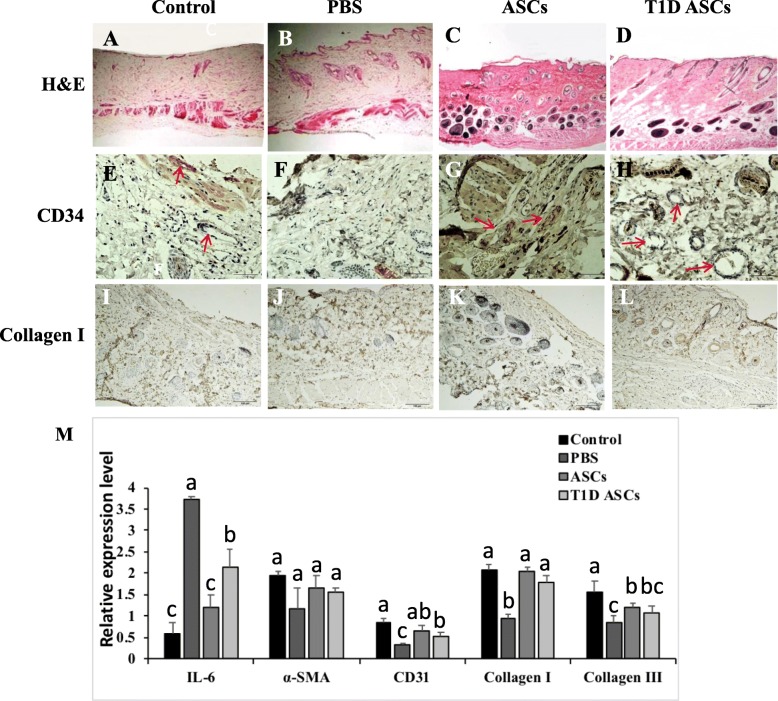
T1D ASCs promote wound vascularity and collagen I expression. H&E staining of wound sections of normal control group treated with PBS (**A**), T1D mice wounds treated with PBS (**B**), ASCs (**C**), or T1D ASCs (**D**) 14 days post-wounding. CD34 (red arrows) immunohistochemistry staining of wound sections of normal control group treated with PBS (**E**); T1D mice wounds treated with PBS (**F**), ASCs (**G**), or T1D ASCs (H) 14 days post-wounding. Collagen I protein immunohistochemistry staining of wound sections of normal control group treated with PBS (**I**); T1D mice wounds treated with PBS (**J**), ASCs (**K**), or T1D ASCs (**L**) 14 days post-wounding. Bars = 100 μm. **M** Gene expression analysis by qRT-PCR using actin as the endogenous control. *p < 0.05, **p < 0.01, ***p < 0.001 vs*.* PBS controls. Triplicate results were averaged and mean ± SD is shown, ANOVA test

To explore the mechanisms of protection of ASCs, we measured formation of subcutaneous glands, epidermal thickness, and follicle formation in regenerated tissue areas in mice receiving T1D or control ASCs (Fig. 4A–D). Newly formed and mature vessels were characterized by CD34 staining at 14 days post-wounding (Fig. 4E–H). These pictures showed that the numbers of newly formed vessels were increased during the healing process for all groups. By staining with the anti-collagen antibody, we found that T1D and control ASC wound showed the higher numbers of newly formed vessels at day 14, compared with T1D treated with PBS (PBS group). As shown in Fig. 4I–L, T1D ASC treatment significantly enhanced re-epithelialization compared to that observed PBS group. Larger collagen deposition areas were observed in the T1D ASC group compared to the untreated group at 14 days post-wounding. We also observed that permutation compactness and uniform distribution of type 1 collagen in T1D ASC and ASC groups.

Inflammation response occurred after cutaneous wounding is a prerequisite for healing. The inflammatory cytokine, IL-6, plays a critical role in wound healing by regulation of leukocyte infiltration, angiogenesis, and collagen accumulation [31]. We found IL-6 expression was reduced in wound treated with T1D ASCs and ASCs (Fig. 3M), suggesting reduced inflammation in ASC treated tissues. However, the amount of IL-6 was higher in T1D ASCs compared to ASCs, suggesting that STZ treatment had a negative impact on the immunomodulation ability of ASCs. Wound healing is also a highly regulated process with α-SMA expression being induced in fibroblast. The increased expression of α-SMA and CD31 is suggesting that the interstitial collagen I and III expression increased in a time-dependent fashion [32]. We measured the mRNA expression of collagen types I and III, α-SMA, and CD31 in scar tissues. Increased collagens I and III were observed in wounds treated with T1D ASCs and control ASCs compared to wounds treated with PBS. Increased CD31 expression in T1D ASC and ASC groups were found when compared to the untreated group at 14 days post-wounding (Fig. 4M).

### T1D ASCs enhance migration and proliferation of fibroblasts

**Fig. 5:**
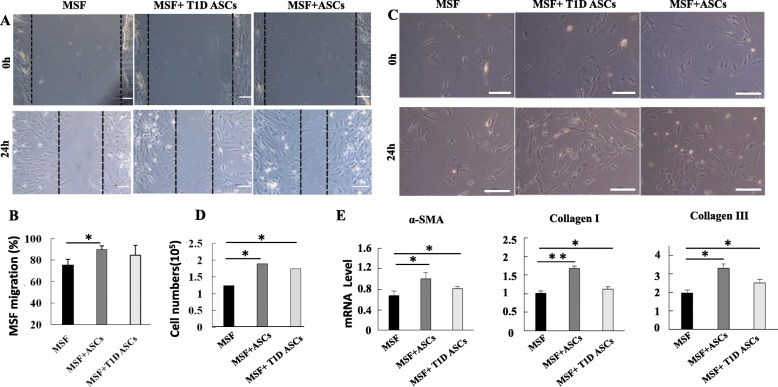
T1D ASCs enhance the migration, proliferation, and α-SMA and collagen I/III expression of fibroblasts in vitro. **A** Representative images of “wounds” in the scratch assay before and 24 h after co-culture with T1D or control ASCs. **B** Migration rates of fibroblasts. **C** Representative images of mice fibroblasts before and 5 days after co-cultured with T1D or control ASCs. **D** Cell numbers of fibroblasts co-cultured with T1D ASCs or ASCs for 5 days. **E** Gene expression of α-SMA, collagen I, and collagen III. Bars = 200 μm. Each bar represents the mean ± SD of three independent experiments. *p < 0.05, **p < 0.01, Student’s *t* test

Migration of fibroblasts to the wound area contributes to wound healing. The effects of ASCs on fibroblasts migration were evaluated by in vitro fibroblast migration assay. Compared to MSF cultured alone, the migration speed of fibroblasts into scratched areas was increased 2.1- and 2.2-folds when co-cultured with T1D or control ASCs (Fig. 5A, B). The proliferation rates of normal mice fibroblasts were also significantly increased after co-culture with T1D or control ASCs (Fig. 5C, D). In addition, mRNA expressions of collagen I, collagen III, and α-SMA were also significantly increased when co-cultured with ASCs (Fig. 5E). There were slightly lower increases of those genes in cells cultured with T1D ASCs compared those with ASCs, although the differences were not significant.

### ASCs can differentiate into vascular endothelial cells and fibroblasts after injection

**Fig. 6:**
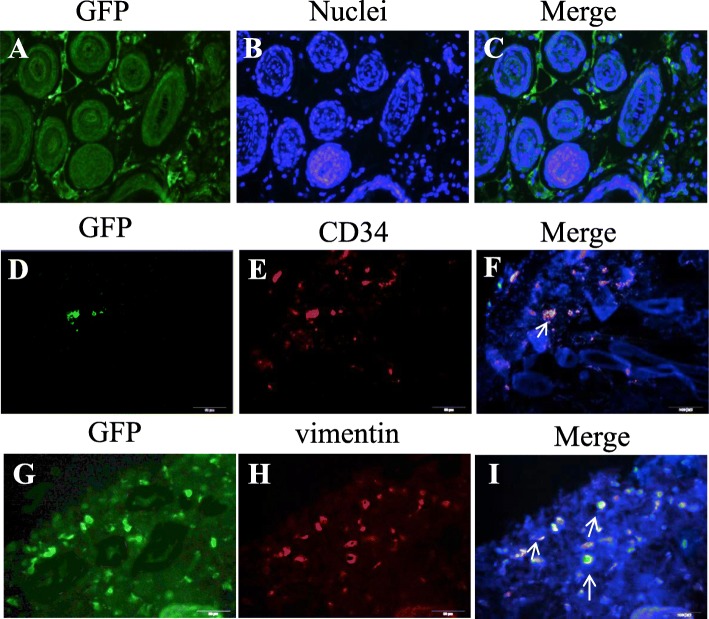
Differentiation of ASCs in mouse wound area. **A**–**C** Representative images show injected GFP-positive ASCs in mice wound. **D** Positive identification of injected ASCs in wound dermal tissue is indicated by GFP staining (green). **E** Red stain indicates CD34. **F** Overlay demonstrates co-localization of GFP and CD34 signal (white arrows). **G** Positive identification of injected ASCs in wound dermal tissue indicated by the GFP staining (green). **H** Red stain indicates vimentin. **I** Overlay demonstrates co-localization of GFP and vimentin signal (white arrows). Bar = 100 μm

To further explore the mechanisms of how ASCs promote wound healing, we tested the differentiation potential of ASCs in vivo, by injecting GFP-positive ASCs in mouse wounds. GFP ASCs were identified by immunohistochemistry using an anti-GFP antibody. Our data showed GFP expression in various substrata of the granulation tissues in the wound area at 2 weeks post-cell infusion (Fig. 6A–C). ASCs were seen incorporating into the winding linear structures along with the newly formed and the mature vessels. GFP and CD34 double-positive cells were observed (Fig. 6D–F). In addition, some injected ASCs displayed a fibroblastic phenotype, based on the GFP/vimentin co-staining at 2 weeks after cell injection (Fig. 6G–I). Our results indicate that ASCs might have contributed to wound healing by differentiation into vascular endothelial cells and fibroblasts in vivo.

## Discussion

The purpose of the present study was to evaluate the capacity of diabetic ASCs in promoting cutaneous wound healing in diabetic mice and also explore the possible mechanisms involved. In this study, we generated mice with hyperglycemia by STZ injection. We referred these mice “T1D” mice although we realized that there are no autoimmune activations in these mice while they all had high blood glucose levels. We found that T1D ASCs and control ASCs showed comparable abilities to improve wound healing in diabetic mice. There was no dramatic morphologic difference between diabetic ASCs and healthy ASCs, nor did the expression of stem cell surface markers including CD29, CD105, and CD34. Several studies have demonstrated that ASC-conditioned medium promotes proliferation and collagen synthesis in human fibroblasts, and ASCs secrete growth factors such as bFGF, TGF-β, and VEGF that play an important role in controlling cell activities at the wound site [33]. It was reported that diabetic ASCs showed reduced proliferation and released lower amounts of growth factors at passage 1 [27]. In these experiments, we found that diabetic ASCs grow slower than nondiabetic ASCs at the first couple of passages. But at passage 3, there was no difference in cell growth rate between these two types of cells. We also found that diabetic ASCs secreted similar amounts of VEGF, bFGF, and TGF-β compared with the normal ASCs at passage 3. It is reasonable to assume that the proliferation and growth factor release function of T1D ASCs were recovered during in vitro cell culture.

Proliferation and migration of fibroblasts play important roles during cutaneous wound healing [34]. Our studies showed that when co-cultured with fibroblasts, diabetic and control ASCs both promoted the proliferation and migration of mice fibroblasts to the same extent, although fibroblasts expressed less collagen and α-smooth muscle actin (α-SMA) when co-cultured with diabetic ASCs.

It was reported that ASCs accelerated diabetic skin wound healing, at least in part through increased collagen synthesis and neovascularization at wound sites [35]. In our study, we found that diabetic ASCs and control ASCs could promote closure of wound in T1D mice, by promoting angiogenesis and increased collagen expression.

One mechanism of ASCs to initiate tissue regeneration is through differentiating into skin cells [36]. In order to determine whether ASCs can differentiate into other cells critical for wound healing in vivo, GFP^+^ASCs were injected into cutaneous wound. Two weeks later, we collected the granulation tissue, stained with fluorescent-conjugated CD34, GFP, and vimentin antibody. Co-staining of GFP/CD34 and GFP/vimentin were observed in some cells. It seems that ASCs differentiated into fibroblasts and endothelial cells in mice. This is consistent with the data reported by Nie et al. [37] and Huang et al. [38].

In our experiments, T1D ASCs and control ASCs showed similar abilities in improving cutaneous wound healing. However, we found that there were still some differences between normal ASCs and T1D ASCs. For example, T1D ASCs need more time to reach 90% confluence, although they did not show growth rate difference after several passages. Less TGF-β secretion, higher IL-6 expression in treated cutaneous wound, and less collagen and α-SMA mRNA expression in co-cultured fibroblasts were observed in T1D ASCs. These differences might have contributed to a slightly less effectiveness in the wound healing rate when compared to healthy ASCs at day 7 (wound closed 18 ± 3.4% and 20 ± 3.2% respectively). The mechanisms behind these differences are still under investigation. In conclusion, although T1D ASCs showed some difference when compared to control ASCs, they still possess significant wound healing properties and might be used for further therapy.

## Conclusions

The results suggest that T1D ASCs could accelerate cutaneous wound healing. The mechanisms may include increasing fibroblast growth and migration, skin angiogenesis, and differentiation into fibroblasts and endothelial cells. These findings provide evidence that diabetic ASCs may be used as a therapeutic option in cutaneous wound healing in diabetic recipients.

## Supplementary information

**Additional file 1: Figure S1.** Representative cell morphology of T1D ASCs (**A**) and control ASCs (**B**) in early passage (bar = 200 μm).

## Data Availability

All data generated or analyzed during this study are included in this published article (and its supplementary information files).

## Abbreviations

ASC: Adipose tissue-derived stem cells
bFGF: Basic fibroblast growth factor
BM-MSCs: Bone marrow-derived mesenchymal stem cells
ELISA: Enzyme-linked immunosorbent assay
FBS: Fetal bovine serum
GFP: Green fluorescent protein
H&E: Hematoxylin and eosin
IL-6: Interleukin-6
InsR: Insulin receptor
ITT: Insulin tolerance test
MSC: Mesenchymal stem cell
MSFs: Mouse skin fibroblast
PBS: Phosphate-buffered saline
RT-PCR: Reverse transcription-polymerase chain reaction
STZ: Streptozotocin
T1D: Type 1 diabetes
TGF-β: Transforming growth factor beta
VEGF: Vascular endothelial growth factor
α-SMA: α-Smooth muscle actin

## References

[1] Tsourdi E, Barthel A, Rietzsch H, Reichel A, Bornstein SR (2013). Current aspects in the pathophysiology and treatment of chronic wounds in diabetes mellitus. Biomed Res Int.

[2] Stadelmann WK, Digenis AG, Tobin GR (1998). Physiology and healing dynamics of chronic cutaneous wounds. Am J Surg.

[3] Chantelau EA (2015). Nociception at the diabetic foot, an uncharted territory. World J Diabetes.

[4] Fahey TJ, Sadaty A, Jones WG, Barber A, Smoller B, Shires GT (1991). Diabetes impairs the late inflammatory response to wound healing. J Surg Res.

[5] Peplow PV, Baxter GD (2012). Gene expression and release of growth factors during delayed wound healing: a review of studies in diabetic animals and possible combined laser phototherapy and growth factor treatment to enhance healing. Photomed Laser Surg.

[6] Browning AC, Alibhai A, McIntosh RS, Rotchford AP, Bhan A, Amoaku WM (2005). Effect of diabetes mellitus and hyperglycemia on the proliferation of human Tenon's capsule fibroblasts: implications for wound healing after glaucoma drainage surgery. Wound repair and regeneration: official publication of the Wound Healing Society [and] the European Tissue Repair. Society..

[7] Okonkwo Uzoagu, DiPietro Luisa (2017). Diabetes and Wound Angiogenesis. International Journal of Molecular Sciences.

[8] Black E, Vibe-Petersen J, Jorgensen LN, Madsen SM, Agren MS, Holstein PE (2003). Decrease of collagen deposition in wound repair in type 1 diabetes independent of glycemic control. Arch Surg.

[9] Naves CC (2016). The diabetic foot: a historical overview and gaps in current treatment. Advances in wound care.

[10] Jones SM, Banwell PE, Shakespeare PG (2005). Advances in wound healing: topical negative pressure therapy. Postgrad Med J.

[11] Stanirowski PJ, Wnuk A, Cendrowski K, Sawicki W (2015). Growth factors, silver dressings and negative pressure wound therapy in the management of hard-to-heal postoperative wounds in obstetrics and gynecology: a review. Arch Gynecol Obstet.

[12] Dalla Paola L, Faglia E (2006). Treatment of diabetic foot ulcer: an overview strategies for clinical approach. Curr Diabetes Rev.

[13] Zahorec P, Koller J, Danisovic L, Bohac M (2015). Mesenchymal stem cells for chronic wounds therapy. Cell Tissue Bank.

[14] Wu Yaojiong, Wang JianFei, Scott Paul G., Tredget Edward E. (2007). Bone marrow-derived stem cells in wound healing: a review. Wound Repair and Regeneration.

[15] Wagner W, Wein F, Seckinger A, Frankhauser M, Wirkner U, Krause U (2005). Comparative characteristics of mesenchymal stem cells from human bone marrow, adipose tissue, and umbilical cord blood. Exp Hematol.

[16] Stolzing A, Jones E, McGonagle D, Scutt A (2008). Age-related changes in human bone marrow-derived mesenchymal stem cells: consequences for cell therapies. Mech Ageing Dev.

[17] Katsara O, Mahaira LG, Iliopoulou EG, Moustaki A, Antsaklis A, Loutradis D (2011). Effects of donor age, gender, and in vitro cellular aging on the phenotypic, functional, and molecular characteristics of mouse bone marrow-derived mesenchymal stem cells. Stem Cells Dev.

[18] Zuk PA, Zhu M, Mizuno H, Huang J, Futrell JW, Katz AJ (2001). Multilineage cells from human adipose tissue: implications for cell-based therapies. Tissue Eng.

[19] Lin K, Matsubara Y, Masuda Y, Togashi K, Ohno T, Tamura T (2008). Characterization of adipose tissue-derived cells isolated with the Celution system. Cytotherapy..

[20] Gimble JM, Bunnell BA, Chiu ES, Guilak F (2011). Concise review: adipose-derived stromal vascular fraction cells and stem cells: let’s not get lost in translation. Stem Cells.

[21] Kato Y, Iwata T, Morikawa S, Yamato M, Okano T, Uchigata Y (2015). Allogeneic transplantation of an adipose-derived stem cell sheet combined with artificial skin accelerates wound healing in a rat wound model of type 2 diabetes and obesity. Diabetes..

[22] Kim WS, Park BS, Sung JH, Yang JM, Park SB, Kwak SJ (2007). Wound healing effect of adipose-derived stem cells: a critical role of secretory factors on human dermal fibroblasts. J Dermatol Sci.

[23] Gao L, Bird AK, Meednu N, Dauenhauer K, Liesveld J, Anolik J (2017). Bone marrow-derived mesenchymal stem cells from patients with systemic lupus erythematosus have a senescence-associated secretory phenotype mediated by a mitochondrial antiviral signaling protein-interferon-beta feedback loop. Arthritis & rheumatology.

[24] Sun LY, Zhang HY, Feng XB, Hou YY, Lu LW, Fan LM (2007). Abnormality of bone marrow-derived mesenchymal stem cells in patients with systemic lupus erythematosus. Lupus..

[25] Koci Z, Turnovcova K, Dubsky M, Baranovicova L, Holan V, Chudickova M (2014). Characterization of human adipose tissue-derived stromal cells isolated from diabetic patient's distal limbs with critical ischemia. Cell Biochem Funct.

[26] Cramer C, Freisinger E, Jones RK, Slakey DP, Dupin CL, Newsome ER (2010). Persistent high glucose concentrations alter the regenerative potential of mesenchymal stem cells. Stem Cells Dev.

[27] Cianfarani Francesca, Toietta Gabriele, Di Rocco Giuliana, Cesareo Eleonora, Zambruno Giovanna, Odorisio Teresa (2013). Diabetes impairs adipose tissue-derived stem cell function and efficiency in promoting wound healing. Wound Repair and Regeneration.

[28] El-Ftesi S, Chang EI, Longaker MT, Gurtner GC (2009). Aging and diabetes impair the neovascular potential of adipose-derived stromal cells. Plast Reconstr Surg.

[29] Fang Q, Zhai M, Wu S, Hu X, Hua Z, Sun H (2019). Adipocyte-derived stem cell-based gene therapy upon adipogenic differentiation on microcarriers attenuates type 1 diabetes in mice. Stem Cell Res Ther.

[30] Cardinal M, Eisenbud DE, Phillips T, Harding K (2008). Early healing rates and wound area measurements are reliable predictors of later complete wound closure. Wound repair and regeneration: official publication of the Wound Healing Society [and] the European Tissue Repair. Society..

[31] Lin ZQ, Kondo T, Ishida Y, Takayasu T, Mukaida N (2003). Essential involvement of IL-6 in the skin wound-healing process as evidenced by delayed wound healing in IL-6-deficient mice. J Leukoc Biol.

[32] Oono T, Specks U, Eckes B, Majewski S, Hunzelmann N, Timpl R (1993). Expression of type VI collagen mRNA during wound healing. J Invest Dermatol.

[33] Shi R, Jin Y, Cao C, Han S, Shao X, Meng L (2016). Localization of human adipose-derived stem cells and their effect in repair of diabetic foot ulcers in rats. Stem Cell Res Ther.

[34] Darby IA, Laverdet B, Bonte F, Desmouliere A (2014). Fibroblasts and myofibroblasts in wound healing. Clin Cosmet Investig Dermatol.

[35] Hu L, Wang J, Zhou X, Xiong Z, Zhao J, Yu R (2016). Exosomes derived from human adipose mensenchymal stem cells accelerates cutaneous wound healing via optimizing the characteristics of fibroblasts. Sci Rep.

[36] Hassan WU, Greiser U, Wang W (2014). Role of adipose-derived stem cells in wound healing. Wound Repair Regen.

[37] Nie C, Yang D, Xu J, Si Z, Jin X, Zhang J (2011). Locally administered adipose-derived stem cells accelerate wound healing through differentiation and vasculogenesis. Cell Transplant.

[38] Huang SP, Huang CH, Shyu JF, Lee HS, Chen SG, Chan JY (2013). Promotion of wound healing using adipose-derived stem cells in radiation ulcer of a rat model. J Biomed Sci.

