# Supplementary figures and images for: Adipose stem cells isolated from diabetic mice improve cutaneous wound healing in streptozotocin-induced diabetic mice
Source: Stem Cell Res Ther. 2020 Mar 17;11:120. doi: 10.1186/s13287-020-01621-x (PMC7079496; doi:10.1186/s13287-020-01621-x)

## Slide 1
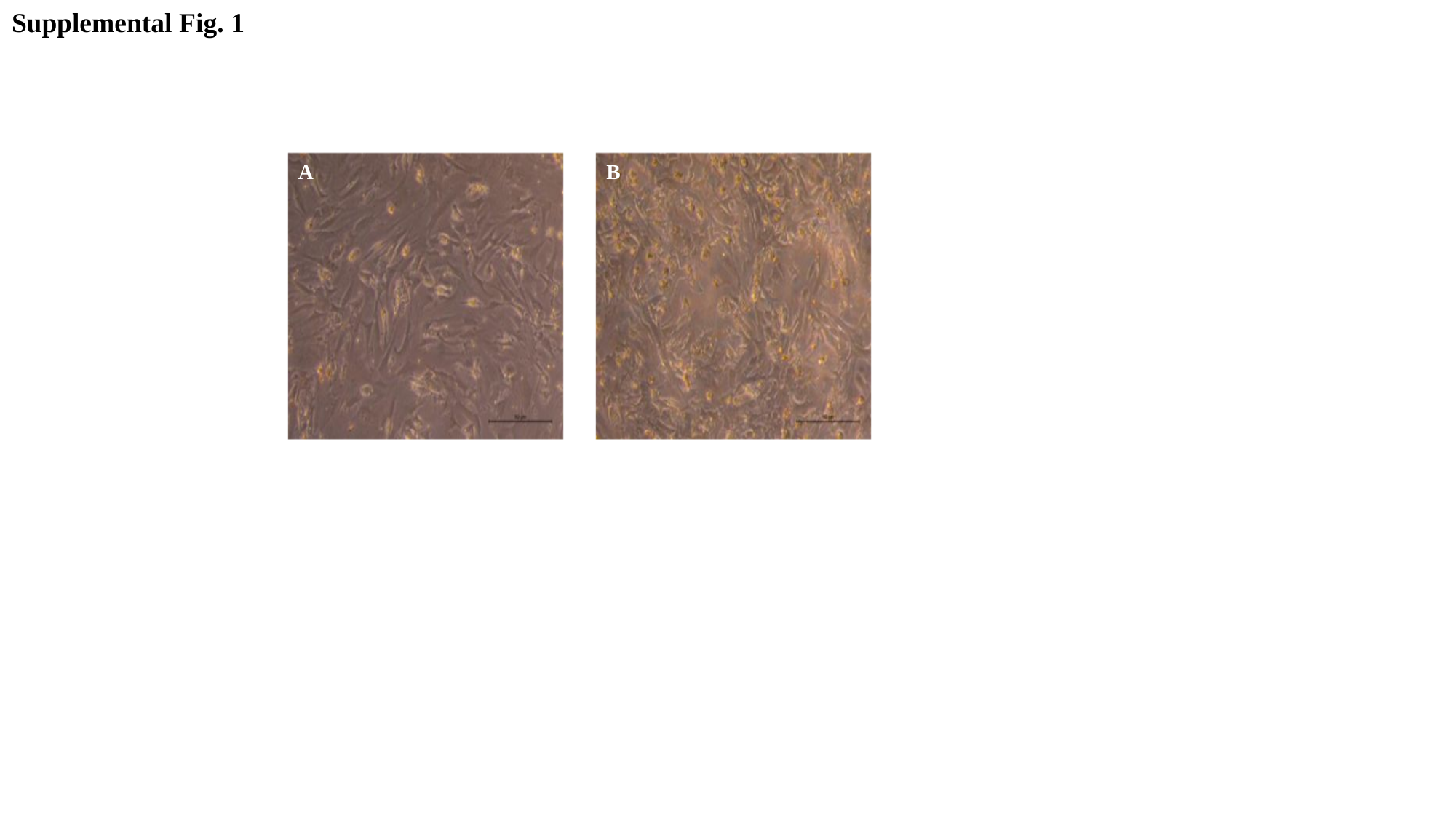

Supplemental Fig. 1
A
B

Supplement: Supplementary file 1 — Additional file 1: Figure S1. Representative cell morphology of T1D ASCs (A) and control ASCs (B) in early passage (bar = 200 μm). [file 13287_2020_1621_MOESM1_ESM.ppt]
